# ﻿Letter for Qiu et al. (2021) regarding ‘The distribution and behavioral characteristics of plateau pikas (*Ochotonacurzoniae*)’

**DOI:** 10.3897/zookeys.1102.79148

**Published:** 2022-05-20

**Authors:** Wen-Jing Li

**Affiliations:** 1 Key Laboratory of Adaptation and Evolution of Plateau Biota, Northwest Institute of Plateau Biology, Chinese Academy of Sciences, Xining, China Key Laboratory of Adaptation and Evolution of Plateau Biota, Northwest Institute of Plateau Biology, Chinese Academy of Sciences Xining China; 2 Scientific Research and Popularization Base of Qinghai-Tibet Plateau Biology, Qinghai Provincial Key Laboratory of Animal Ecological Genomics, Xining, China Scientific Research and Popularization Base of Qinghai-Tibet Plateau Biology, Qinghai Provincial Key Laboratory of Animal Ecological Genomics Xining China

## ﻿

Dear Editor,

While reading the paper ‘The distribution and behavioral characteristics of plateau pikas (*Ochotonacurzoniae*)’ (Qiu et al. 2021), I found that the authors of this paper appear to have made a mistake in reporting the surface temperature of their research area. The reasons are as follows:

The study was carried out in August 2019 in Dari County of Qinghai Province, China (Qiu et al. 2021). I would like to point out that the historic high air temperature in Dari County was 23.2 °C (Wang et al. 2018), but in the paper by Qiu et al. (2021), the highest surface temperature was reported as 48 °C, and was more than twice the historic high temperature recorded for their research area. The reason for this discrepancy was that the surface temperature recorded in images was the temperature that was detected by the temperature receptors of the field infrared camera. There was no shelter provided for the field infrared camera in alpine meadow grasslands and the temperature of the camera increased rapidly under direct sunshine. However, the authors in this study incorrectly used the temperature recorded by the camera directly and the values shown for the temperature gradient in figure 6 of Qiu et al. (2021) were out of the range of the normal air temperature conditions. Therefore, their conclusion that the preferable temperature for pikas may be around 31~35 °C was incorrect. The altitude of the distribution areas of plateau pikas is more than 3,000 m, and cool condition are preferred by the pikas; if the temperature is more than 25 °C, the pikas will die. Therefore, I suggest that the authors of Qiu et al. (2021) provide a statement of their results about temperature and behavior, or re-analyze of the data according to the temperature from the Meteorological Bureau of Dari County.

This is a common problem in research projects that use temperature recordings recorded directly from images from field infrared cameras, which often produce incorrect air temperature readings when the cameras are under direct sunshine. Therefore, I suggest that if the temperature recorded by the camera is to be used in the research, that the camera be housed in a proper instrument weather shelter, or that the temperature be recorded or obtained separately.
